# Opportunities for using spatial property assessment data in air pollution exposure assessments

**DOI:** 10.1186/1476-072X-4-26

**Published:** 2005-10-31

**Authors:** Eleanor M Setton, Perry W Hystad, C Peter Keller

**Affiliations:** 1 Spatial Sciences Research Laboratory, Geography Department, University of Victoria, PO BOX 3050 STN CSC, Victoria, B.C., V8W 3P5, Canada

## Abstract

**Background:**

Many epidemiological studies examining the relationships between adverse health outcomes and exposure to air pollutants use ambient air pollution measurements as a proxy for personal exposure levels. When pollution levels vary at neighbourhood levels, using ambient pollution data from sparsely located fixed monitors may inadequately capture the spatial variation in ambient pollution. A major constraint to moving toward exposure assessments and epidemiological studies of air pollution at a neighbourhood level is the lack of readily available data at appropriate spatial resolutions. Spatial property assessment data are widely available in North America and may provide an opportunity for developing neighbourhood level air pollution exposure assessments.

**Results:**

This paper provides a detailed description of spatial property assessment data available in the Pacific Northwest of Canada and the United States, and provides examples of potential applications of spatial property assessment data for improving air pollution exposure assessment at the neighbourhood scale, including: (1) creating variables for use in land use regression modelling of neighbourhood levels of ambient air pollution; (2) enhancing wood smoke exposure estimates by mapping fireplace locations; and (3) using data available on individual building characteristics to produce a regional air pollution infiltration model.

**Conclusion:**

Spatial property assessment data are an extremely detailed data source at a fine spatial resolution, and therefore a source of information that could improve the quality and spatial resolution of current air pollution exposure assessments.

## Background

Many epidemiological studies examining the relationships between adverse health outcomes and exposure to air pollutants use ambient air pollution measurements as a proxy for personal exposure levels [1]. Because the number of fixed outdoor monitoring sites within a city usually is limited, ambient pollution measurements often are extrapolated to areas between monitors, thus disregarding any neighbourhood-scale spatial variation in pollution levels. Recent research suggests that some neighbourhoods within a city can be disproportionately exposed to air pollution and that these differences may influence health outcomes [2]. A major constraint to moving toward exposure assessments and epidemiological studies of air pollution at a neighbourhood level is the lack of readily available data at appropriate spatial resolutions.

Spatial property assessment data (SPAD) were identified as a potential data source for exposure research through an ongoing study, funded by Health Canada via the British Columbia Centre for Disease Control, examining the effects of air pollution on birth outcomes and subsequent development of health outcomes associated with exposure to air pollution for a birth cohort of 90,000. The study area encompasses the Georgia Basin Puget Sound airshed, located in the Pacific Northwest of the United States and Canada and encompassing approximately 10 million hectares of land and marine environments. SPAD (considered here to be made up of both tabular assessment data on building characteristics and spatial data that show the location of each property) for every assessed parcel of land are generally available for the airshed as spatially referenced digital databases, suitable for use with common geographic information systems (GIS). These data may increase the resolution and accuracy of variables used in exposure assessment models and epidemiological analyses, but the authors have found few published studies using SPAD for developing exposure assessments or in epidemiological analyses of air pollution impacts on health.

The purpose of this paper is to describe SPAD and to illustrate their potential utility for neighbourhood level exposure estimates and epidemiological research. Three possible uses of SPAD are examined, including: (1) creating variables for use in land use regression modelling of neighbourhood levels of ambient air pollution; (2) enhancing wood smoke exposure estimates by mapping fireplace locations; and (3) using data available on individual building characteristics to produce a regional air pollution infiltration model.

## Results and discussion

### Typical SPAD characteristics

Tabular assessment data generally incorporate two kinds of information: 1) property addresses or property identifiers that can be used for spatial referencing in conjunction with digital street networks or digital cadastral maps; and 2) descriptive variables including building characteristics, building and land values, and land use information on which annual property tax assessments are based. Table 1 provides examples of common variables in tabular assessment data that may be important to air pollution exposure assessments and epidemiological analyses.

**Table 1:** Common variables in tabular assessment data

**Location**	School District #, Area #, Township Range, Jurisdiction #, Neighbourhood #, Street Address, Street Direction, Street Type, ZIP Code, City, Property Identifier
**Land**	Appraisal Date, Property Size, Property Use Code, Land Use Code, Electricity, Water, Sewer, Street Surface Type, # of Dwelling Units, # of Outbuildings, # of Improvements, Building Permit.
**Sales**	Sale Date, Sale Price, Sales Excise Number, Deed Type, Qualification Code, Multiple Sales, Land Value, Improvements Value.
**Improvements**	Improvement Type, Structure Use, Building Type, # of Stories, Year Built, Total Square Footage, # of Bedrooms, Predominant Heating Type, Fireplace, Structural Quality.

SPAD are created when tabular property assessment data are spatially referenced, either by linking property addresses to a digital street network, or by linking property identifiers to a digital cadastral map. Spatial referencing allows for complex queries of the tabular assessment, the results of which can be mapped. In effect, the spatial resolution of SPAD is the individual parcel size, generally much finer than other spatially referenced data commonly used in exposure assessment and epidemiological analyses, such as Census data.

### Data format and availability

**Figure 1:**
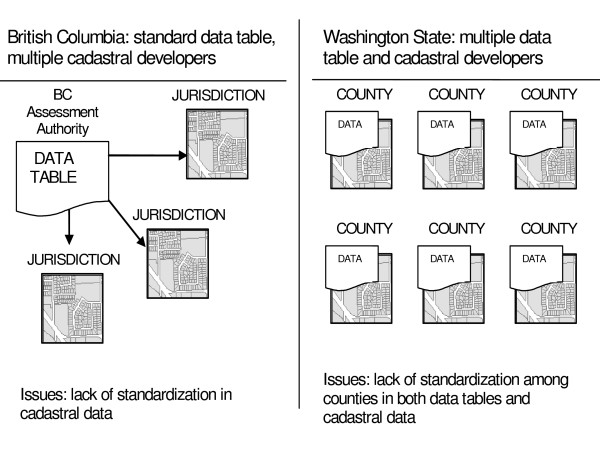
The development of SPAD differs between British Columbia and Washington State.

SPAD are developed and maintained by numerous jurisdictions throughout North America, therefore data format and content may differ significantly among jurisdictions. The following discussion highlights some of the differences and associated issues in British Columbia and Washington State, as shown in Figure 1. In British Columbia, tabular assessment data are collected by the BC Assessment Authority and maintained as a tabular database, while each taxing municipality or regional district develops its own cadastral data that can be linked with assessment data to create SPAD. By agreement, BC Assessment uses a unique property identifier for each assessment record, and the same property identifiers are used by taxing jurisdictions when developing cadastral data, thereby enabling a linkage between the tabular assessment data from BC Assessment and the jurisdiction's cadastral data using GIS. In Washington State, each county is responsible for developing both the tabular assessment data and the cadastral data. In this regard, there is some advantage in the British Columbia system, in that one single authority collects and maintains the tabular assessment data and so these are standard throughout the entire province. Unfortunately, when multiple jurisdictions are responsible for developing tabular assessment data and/or cadastral data, there can be significant differences in format among jurisdictions. For example, in British Columbia, cadastral data are often available in ESRI^© ^GIS formats, but in some cases are only available in AutoCad^© ^format. The latter is primarily an engineering and drafting application and the format may not always translate easily into GIS formats. In Washington State, neither the tabular assessment data nor the cadastral data may be standardized among counties, as each develop and maintain their own information systems. In this case, it is possible that some tabular assessment data (i.e., presence of air conditioner) are collected for one county, but not for the adjacent county, or that different GIS applications are used by different counties.

Access to SPAD (or its constituent tabular and cadastral data) is markedly different in British Columbia in comparison to Washington State. In British Columbia, researchers must negotiate data sharing or purchasing agreements with each jurisdiction in order to access SPAD, and may also have to purchase additional tabular assessment data directly from the BC Assessment Authority in order to develop SPAD specific to the research question. In Washington State, SPAD are available for download through each county's internet site, or may be ordered directly from each county at no cost or for a small fee (i.e., for CD writing and postage). In many cases, due to large file sizes, the tabular assessment data and the spatial cadastral data are provided separately, and must be linked by the researcher using GIS to create the final SPAD.

Linking tabular assessment data using property addresses or identifiers to produce SPAD is not always trouble free. In cases where tabular assessment data and the spatial cadastral data are provided by the same jurisdiction, linking the two datasets often is easily accomplished. In Washington State, for example, where each county develops and maintains its own SPAD, we were able to download the tabular assessment data and the spatial cadastral data, and link each record with a 98 percent success rate. For the British Columbia portion of the airshed, we initially purchased tabular assessment data from the BC Assessment Authority and spatially referenced them using the included property addresses and a commercially available digital street network with ESRI^© ^ArcGIS 8.1. Approximately 1.1 million records were received from BC Assessment for the entire Georgia Basin airshed, which is comprised of 26 separate taxing jurisdictions. Linking between the tabular assessment data and the street network was successfully completed for approximately 83 percent of the records, with the number of links in urban areas better than in rural regions (89 percent versus 67 percent respectively). The lower success rate in rural regions is generally due to incomplete or non-standard street addresses (i.e., post office boxes or rural post offices rather than street addresses) in the tabular assessment data. Also, the road network (circa 2003) did not contain information on the most recent subdivisions and new construction, so those properties were excluded by default. We subsequently acquired cadastral data from each of the 26 taxing authorities, and achieved an average success rate of 96 percent when linking the tabular data provided by BC Assessment Authority. Obviously, linking tabular assessment data to cadastral data is preferred; however, in jurisdictions without digital cadastral data, using a digital street network may be the only option, and link success rates may vary widely.

### Developing variables from SPAD for use in land use regression models of neighbourhood pollution levels

When adequate measured data are not available, neighbourhood level exposure assessments may use outdoor pollution levels derived by models that require land use data as inputs. For example, land use regression (LUR) models have been used to predict traffic-related air pollution levels for neighbourhood areas depending on nearby roads, traffic volume, population density, and land uses [3-5]; these predicted levels were then used as indicators of exposure for epidemiological analyses. In their 1997 study, Briggs, Collins et al. used land cover data interpreted from aerial photographs, as well as building density (six classes) derived from local planning maps in a LUR model to predict spatial surfaces of nitrogen dioxide (NO_2_) levels in three European cities [4]. In 2003, Brauer et al. used 100 m raster grids of population density in a LUR model to predict fine particulate (PM_2.5_) levels at over 10,000 residential addresses in Sweden and the Netherlands [5]. The 100 m raster grids of population density were developed by national agencies from population registries that record the current residential address for most of the population. In research currently underway in the Pacific Northwest, Brauer, Henderson, et al. have included the area of commercial land, provided by local government as a digital map, as a predictor in a LUR model of traffic-related air pollution in Vancouver, British Columbia [6].

**Figure 2:**
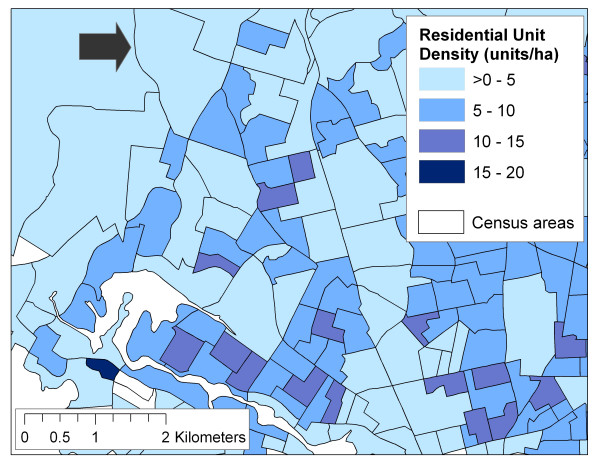
Residential unit density reported for census areas.

**Figure 3:**
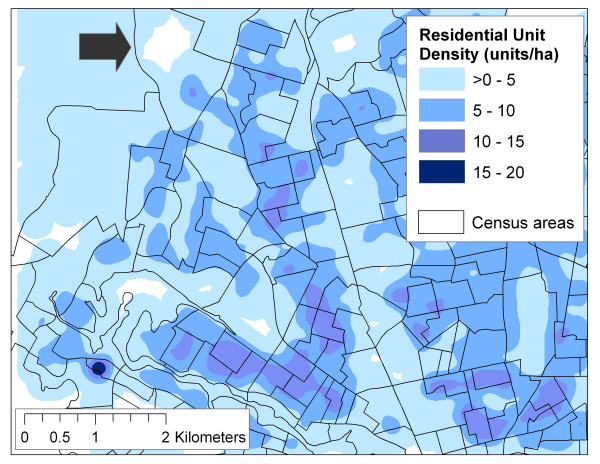
Residential units density surface calculated using a GIS kernel function.

SPAD provide a unique opportunity to develop neighbourhood-level variables for use in LUR models. Whereas developing land use data from air photo interpretation or local planning maps may not be feasible for large study areas, there are no standard population registries in North America, and local digital land use maps may not be readily available, SPAD can be used to develop variables measuring building density, population density, residential unit density, and commercial land use (among others). In fact, SPAD may present an opportunity to significantly improve the spatial resolution of these kinds of density measures since SPAD are essentially individual-level data (i.e., available for every parcel), in contrast with widely used census data which are only available pre-aggregated for fixed census areas. Because SPAD are individual-level data, density measures can be based on any area(s) defined by the researcher, rather than restricted to existing census areas which may not adequately define the true areas of interest. Perhaps more importantly, current GIS can easily create spatial surfaces of density given several distance parameters (i.e., calculate density for every 10 m × 10 m cell in the study area, based on the number of residential units within 100 m of the cell centre). Figures 22 and 3 provide an illustration of the improved spatial resolution in measuring density using SPAD. Figure 2 shows residential density per hectare using SPAD, but reported for census boundaries. Note that the large census area near the top of the figure is shown with a residential density of >0 – 5. In Figure 3, residential density per hectare was calculated from SPAD using a GIS kernel function, and shows that the same large census area near the top in fact has a range of residential densities. When making neighbourhood level exposure assessments, variations on the scale of several hundred metres may be important. Note that the area shown in Figures 2 and 3 is approximately 4.8 hectares in size.

SPAD also provide very detailed information about land use. In British Columbia, properties have a designated actual use code, organized in an hierarchical fashion. For example, a property's Level 1 (Property Code) designation may be 'major industry'; the Level 2 (Actual Use Code) designation may be 'primary metal industry', and its Level 3 (Manual Class Code) designation may be 'primary smelting and refining'. Similarly, a residential property may be designated as residential, single family residence, and 1 1/2 storey good condition. In British Columbia, there are 950 unique Level 3 designations. In Washington State, the property use codes used by counties generally correspond to the standard Land Use Coding Manual created by the Urban Renewal Administration, Housing and Home Finance Agency and Bureau of Public Roads (1965), which contains 4 levels of classification (see  for more information).

**Figure 4:**
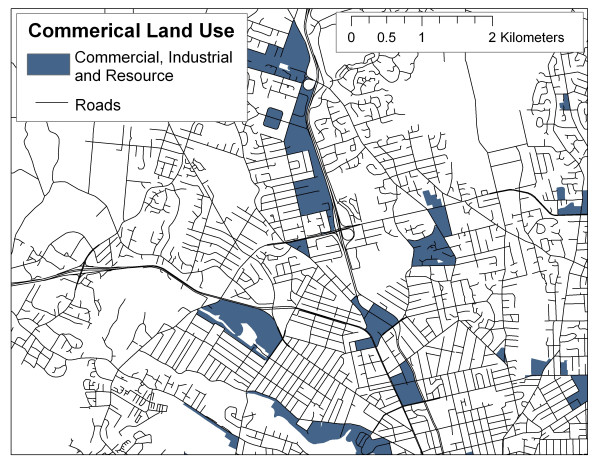
Commercial land use from DMTI Spatial Inc.

**Figure 5:**
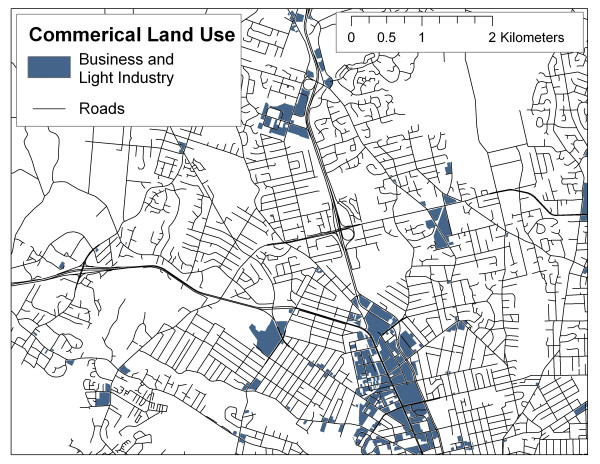
Commercial land use from SPAD.

**Figure 6:**
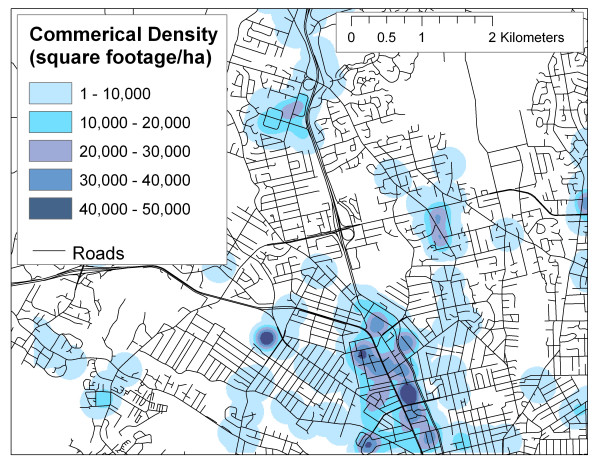
Density of commercial square footage derived from SPAD using a GIS kernel function.

Other sources of land use data are provided in highly generalized formats with pre-defined land use classes which may not be optimal for researchers. For example, DMTI ^© ^Spatial produces a commercially available land use dataset for GIS use, with the following land use classes: commercial, government and institutional, open area, parks and recreational, residential, resource and industry, and waterbody (see  for more information). Figures 4 and 5 illustrate the different spatial distributions of commercial and industrial classes based on DMTI ^© ^Spatial data and SPAD (including properties coded as industrial or business, assuming that these classifications in SPAD are comparable to commercial and resource/industrial classifications in the DMTI ^© ^Spatial land use dataset), suggesting that significantly different results could be obtained for the same LUR model, depending on which data set is employed. This is not to suggest that the DMTI ^© ^Spatial data set is of poor quality, instead, it should be noted that this data set and others like it have been prepared for specific purposes and for use at general spatial scales that may not be adequate for neighbour-level exposure assessment. Also shown, in Figure 6, is a density map (square footage of business and industrial buildings per hectare) based on SPAD. If commercial and industrial activity is meant to act as a surrogate for air pollution, it is argued here that the density map produced with SPAD could provide a much more accurate measure of the level of commercial/industrial activity than do simple land use maps.

What is not readily apparent in Figures 5 and 6 is the high level of additional detail on land use inherent in the SPAD, which can be used to further refine land use classifications. In the above example, parcels from SPAD were selected based on the first level of description, the Property Code. Table 2 provides a summary of the Actual Use Code for all parcels selected and illustrates the wide variety of land uses included in the more general Property Code classification. This additional detail provides significant flexibility to researchers in terms of developing surrogate indicators of ambient air pollution based on land use, as they can include or exclude properties from the indicator based on more refined conceptual links to ambient air pollution. For example, researchers may choose to include parking lots since vehicle use may be concentrated there, but exclude vacant properties as no current activity occurs.

**Table 2:** Detailed information from SPAD for commercial land use

**'Actual Use' Classification**	**Parcels (n)**	**'Actual Use' Classification**	**Parcels (n)**
Storage and Warehousing – closed	100	Department Store	4
Stores and Services – Commercial	97	Fast Food Restaurant	4
Office Building (primary use)	70	Automobile sales – lot	3
Vacant	53	Industrial – Vacant	3
Parking Lot	27	Self-Serve Service Station	3
Commercial – strata lot	25	Shopping Center – neighbourhood	3
Automobile Paint Shop/Garage	23	Food Market	2
Stores and Offices	15	Metal Fabricating Industry	2
Automobile dealership	13	Shopping Center – regional	2
Shopping Center	10	Bakery and Biscuit Manufacturing	1
Shopping Center – community	10	Bowling Alley	1
Convenience Store/Service Station	9	Car Wash	1
Motel and Auto Court	9	Clothing Industry	1
Restaurant	9	Confectionary Manufacturing	1
Lumber Yard or Building Supplies	8	Furniture and Fixtures Industry	1
Service Station	8	Marine and Navigational Facilities	1
Hotel	5	Sash and Door Industry	1
Neighbourhood Pub	5	Soft Drink Bottling	1
Neighbourhood Store	5	Storage and Warehousing – cold	1
Bank	4	Stores and Living Quarters	1
Bus Company	4	Transportation Equipment	1

### Using SPAD to estimate exposure to wood smoke

Exposure to wood smoke has been associated with negative health impacts, particularly for children and the elderly [7-9] and there is increasing interest in developing models to predict spatial estimates of wood smoke levels in order to provide spatially refined estimates that do not rely on individual surveys or monitoring campaigns. Spatial estimates of residential wood burning have been included in regional emissions inventories prepared for air quality management purposes and so a very brief overview of the methods used for emissions inventory purposes is provided here. In general, the contribution of residential wood burning to regional air quality is estimated by applying an emission factor to the proportion of households thought to have a wood burning appliance. Both the emission factor and the proportion of households are often derived from telephone surveys conducted in the region of interest. An example of this approach, employed for eight regions in British Columbia, is described in a recent report produced by the British Columbia Ministry of Water, Land and Air Protection [10]. Recent research by Tian et al. describes an approach in which a number of spatial variables are used to predict the proportion of wood-burning households, similar to the LUR models described above [11]. In their study, Tian et al. found that elevation, age (retired or ages 34-54), presence of farm income, and owner occupied residences predicted the number of households using wood as a primary heating source (as per the 1990 US Census) for census block groups. While it is not clear how this improves on the data already available from the US Census (at least for 1990 and 2000), this method could be used where US Census data do not exist, i.e., Canada.

**Figure 7:**
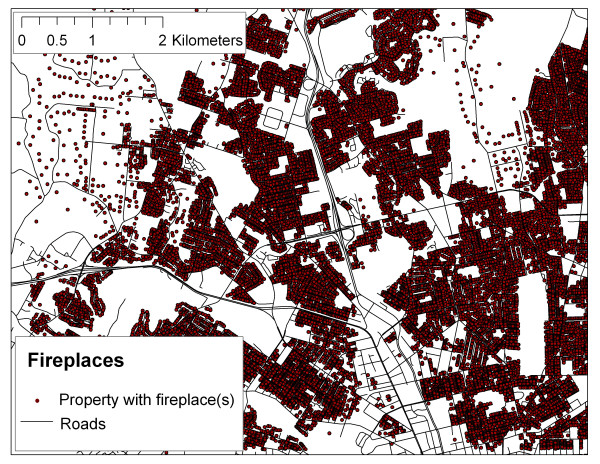
Map of fireplace locations.

**Figure 8:**
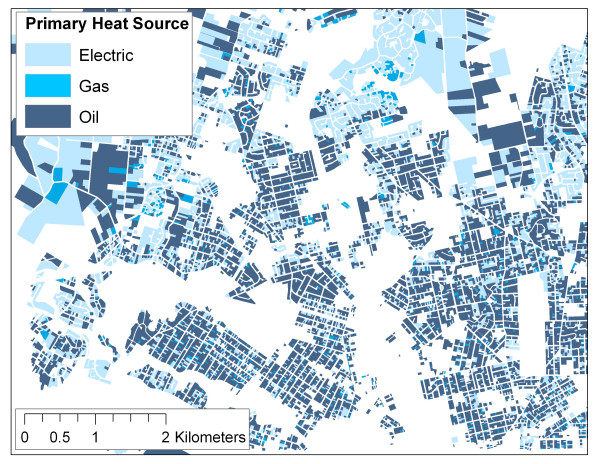
Map of primary heat sources based on SPAD.

Using SPAD, it is possible to locate wood burning appliances, and to map predominant heating source (i.e., electric baseboards, electric radiant, forced hot air, electric forced hot air, gas forced hot air, oil forced hot air, heat pump, hot water, etc.), as shown in Figures 7 and 8. This spatial information provides an opportunity to greatly increase the spatial resolution of wood smoke estimates over those derived from census variables and regional telephone surveys.

In the context of epidemiological studies, Larson et al. have used SPAD in conjunction with other spatial variables in order to predict fine particulate (PM_2.5_) levels associated with wood smoke for a large epidemiological study currently underway in the Georgia Basin Puget Sound Airshed [12]. Preliminary results suggest that building age, population density, and number of fireplaces are relatively strongly correlated with measured PM_2.5 _in the study area. A range of socio-economic variables are more weakly correlated. Of particular interest, this approach negates the need for additional information on wood-burning practices and emissions factors by relating spatial variables derived from SPAD and other sources (i.e., Census data) directly to actual measures of PM_2.5 _on cold clear evenings.

### Infiltration modelling using SPAD

Population level epidemiological studies of air pollution commonly use an indirect approach to exposure assessment by assigning exposure levels based on outdoor ambient air pollution levels at the residential location, even though an increasing number of personal monitoring studies have shown that exposure measurements based on ambient monitoring are usually lower than those derived from personal monitoring [13]. Strong associations have been found between indoor and outdoor PM_2.5 _concentrations which indicate that a significant proportion of indoor fine particles are of outdoor origin [14], and other studies have identified specific building characteristics that influence infiltration rates, for example, type of basement, and year of construction [15].

SPAD contain a variety of information on building characteristics (Table 3) that could be incorporated into a regional infiltration model when used in conjunction with data on external conditions, such as climate factors, wind shielding, wind speed and direction. Such an infiltration model could provide a more complete picture of indoor pollutant levels, the spatial distributions of infiltration rates, and the impacts of indoor exposure to total exposure levels and health outcomes. The authors currently are developing an infiltration model for the Georgia Basin Puget Sound airshed, based on SPAD and an indoor/outdoor PM_2.5 _monitoring program.

**Table 3:** Variables common in SPAD that may be used in a regional infiltration model

**Land Variables**	Property Size, Property Use, Topography, Building Permit Class.
**Building Variables**	Improvement Type, Structure Use, Building Type, # of Stories, Year Built, Total Square Footage, Predominant Construction Type, # of Bedrooms, Predominant Heating Type, Air Conditioning, Fireplace, Structural Quality.

## Conclusion

Considering that many exposure assessments and epidemiological analyses of the impacts of air pollution on health have been undertaken at regional scales, and that only recently have researchers begun to investigate neighbourhood-level variation in pollutant levels, it is not surprising that the authors could not find any published exposure assessments or epidemiological studies of air pollution that made use of SPAD. This paper illustrates that SPAD are a readily available data source that may provide an opportunity for conducting air pollution exposure assessment at neighbourhood level scales. SPAD also provide highly detailed information on building characteristics that may prove useful for modelling indoor levels of ambient-origin air pollution based on building infiltration characteristics, and there may be some utility in using SPAD to develop or refine indicators of socio-economic status. Some limitations to using SPAD are also apparent: SPAD are very large datasets which require GIS software and expertise to clean and extract the required subset of data in order to avoid slow processing times; and issues of comparability between GIS formats and data content may arise when a study area encompasses more than one jurisdiction. Limitations notwithstanding, the authors expect to see increasing uses of SPAD for exposure assessment and epidemiological analyses in the future, as researchers continue to investigate spatial variations in pollutant levels and other factors affecting exposure at increasingly finer scales.

## Methods

SPAD were developed for the Canadian (southwest British Columbia) portion of the airshed by spatially referencing tabular property assessment data provided by the province to cadastral (parcel) data provided by municipal governments. For the American portion of the airshed (a portion of Washington State) the data were acquired in a readily useable format from each county. These data are used to illustrate the typical characteristics of SPAD, and to identify issues for using SPAD in terms of format, attributes and availability. Conceptual applications of SPAD to exposure assessment are demonstrated using SPAD from British Columbia and Washington State.

## Authors' contributions

ES and PH acquired and processed the spatial property assessment data. ES prepared the manuscript with support from PH. PK reviewed and edited the manuscript. All authors read and approved the final manuscript.

## References

[B1] Williams FLR, Ogston SA (2002). Identifying populations at risk from environmental contamination from point sources. Occup Environ Med.

[B2] O'Neill MS, Jerrett M, Kawachi L, Levy JL, Cohen AJ, Gouveia N, Wilkinson P, Fletcher T, Cifuentes L, Schwartz J (2003). Health, wealth, and air pollution: Advancing theory and methods. Environmental Health Perspectives.

[B3] Clench-Aas J, Bartonova A, Bohler T, Gronskei KE, Sivertsen B, Larssen S (1999). Air pollution exposure monitoring and estimating Part I. Integrated air quality monitoring system. Journal of Environmental Monitoring.

[B4] Briggs DJ, Collins S, Elliott P, Fischer P, Kingham S, Lebret E, Pryl K, Van Reeuwijk H, Smallbone K, Van der Veen A (1997). Mapping urban air pollution using GIS: a regression-based approach. International Journal of Geographical Information Science.

[B5] Brauer M, Hoek G, van Vliet P, Meliefste K, Fischer P, Gehring U, Heinrich J, Cyrys J, Bellander T, Lewne M, Brunekreef B (2003). Estimating long-term average particulate air pollution concentrations: Application of traffic indicators and geographic information systems. Epidemiology.

[B6] Brauer M, Henderson S, Jerrett M, Beckerman B (2005). Land Use Regression Modeling of Nitrogen Oxides and Fine Particulate Matter in the Greater Vancouver Regional District: November 8 - 11; Blaine, Washington..

[B7] Salam MT, Li YF, Langholz B, Gilliland FD (2004). Early-life environmental risk factors for asthma: Findings from the children's health study. Environmental Health Perspectives.

[B8] Boman BC, Forsberg AB, Jarvholm BG (2003). Adverse health effects from ambient air pollution in relation to residential wood combustion in modern society. Scandinavian Journal of Work Environment & Health.

[B9] Larson TV, Koenig JQ (1994). Wood Smoke - Emissions and Noncancer Respiratory Effects. Annual Review of Public Health.

[B10] British Columbia Ministry of Water LAP (2004). Residential Wood Burning Emissions in British Columbia.

[B11] Tian YQ, Radke JD, Gong P, Yu Q (2004). Model development for spatial variation of PM2.5 emissions from residential wood burning. Atmospheric Environment.

[B12] Larson T, Su J, Baribeau A, Buzzelli M, Setton E, Brauer M (2005). A Spatial Model of Urban Winter Woodsmoke Concentrations: ; Blaine, Washington..

[B13] Toivola M, Alm S, Reponen T, Kolari S, Nevalainen A (2002). Personal exposures and microenvironmental concentrations of particles and bioaerosols. Journal of Environmental Monitoring.

[B14] Rojas-Bracho L, Suh HH, Oyola P, Koutrakis P (2002). Measurements of children's exposures to particles and nitrogen dioxide in Santiago, Chile. Science of the Total Environment.

[B15] Chang TJ, Huang MY, Wu YT, Liao CM (2003). Quantitative prediction of traffic pollutant transmission into buildings. Journal of Environmental Science and Health Part a-Toxic/Hazardous Substances & Environmental Engineering.

